# Urodele p53 tolerates amino acid changes found in p53 variants linked to human cancer

**DOI:** 10.1186/1471-2148-7-180

**Published:** 2007-09-28

**Authors:** Éric Villiard, Henner Brinkmann, Olga Moiseeva, Frédérick A Mallette, Gerardo Ferbeyre, Stéphane Roy

**Affiliations:** 1 Department of Biochemistry, Faculty of Medicine, Université de Montréal, Montréal, QC, H3C 3J7, Canada; 2 Department of Stomatology, Faculty of Dentistry, Université de Montréal, Montréal, QC, H3C 3J7, Canada

## Abstract

**Background:**

Urodele amphibians like the axolotl are unique among vertebrates in their ability to regenerate and their resistance to develop cancers. It is unknown whether these traits are linked at the molecular level.

**Results:**

Blocking p53 signaling in axolotls using the p53 inhibitor, pifithrin-α, inhibited limb regeneration and the expression of p53 target genes such as Mdm2 and Gadd45, suggesting a link between tumor suppression and regeneration. To understand this relationship we cloned the p53 gene from axolotl. When comparing its sequence with p53 from other organisms, and more specifically human we observed multiple amino acids changes found in human tumors. Phylogenetic analysis of p53 protein sequences from various species is in general agreement with standard vertebrate phylogeny; however, both mice-like rodents and teleost fishes are fast evolving. This leads to long branch attraction resulting in an artefactual basal emergence of these groups in the phylogenetic tree. It is tempting to assume a correlation between certain life style traits (e.g. lifespan) and the evolutionary rate of the corresponding p53 sequences. Functional assays of the axolotl p53 in human or axolotl cells using p53 promoter reporters demonstrated a temperature sensitivity (ts), which was further confirmed by performing colony assays at 37°C. In addition, axolotl p53 was capable of efficient transactivation at the Hmd2 promoter but has moderate activity at the p21 promoter. Endogenous axolotl p53 was activated following UV irradiation (100 j/m^2^) or treatment with an alkylating agent as measured using serine 15 phosphorylation and the expression of the endogenous p53 target Gadd45.

**Conclusion:**

Urodele p53 may play a role in regeneration and has evolved to contain multiple amino acid changes predicted to render the human protein defective in tumor suppression. Some of these mutations were probably selected to maintain p53 activity at low temperature. However, other significant changes in the axolotl proteins may play more subtle roles on p53 functions, including DNA binding and promoter specificity and could represent useful adaptations to ensure p53 activity and tumor suppression in animals able to regenerate or subject to large variations in oxygen levels or temperature.

## Background

Inactivation of p53 by mutations or viral oncogenes is the most frequent alteration found in human cancers [1]. P53 counteracts the process of neoplastic transformation by preventing the proliferation of cells with genomic abnormalities [1]. Multiple stress conditions activate p53 including DNA damage, hypoxia, redox stress, ribonucleotide imbalance, cell adhesion and oncogenes [2-5]. In response to these signals, p53 undergoes a variety of post-translational modifications, such as phosphorylation, acetylation and sumolation, which modulate its stability and activity [5]. The effects of p53 are mediated through the induction of a variety of genes that have not yet been fully characterized. These genes induce transient cell cycle arrest, permanent cell cycle arrest program (senescence) or a cell death program (apoptosis) [1,6].

Most of the research trying to resolve the function of p53 has been accomplished on transformed cells. However, cell culture experiments represent only a limited perspective of the non-autonomous function of p53 as it occurs in whole organisms. Hence, the function of p53 beyond that observed in isolated cells remains largely a black box. Needless to say, that the actual role of p53 in vivo is not well understood. For these reasons researchers have turned to the mouse as an in vivo model system to study p53 functions [7]. The mouse system circumvents many of the problems associated with the use of cultured cells to study p53, but fails to model the human condition in a number of important issues. One critical difference is the short life span exhibited by laboratory mice. Longevity in humans imposes a high selective pressure to develop and refine tumor suppression pathways that might be better studied in other long living animal models. In addition, p53 null mice are surprisingly normal [8]. The longevity factor is also of importance considering the ability of p53 to promote aging in mice even while increasing cancer protection [9]. For these reasons, knowledge of the p53 pathway in other animal models may contribute critical insights into its biological functions.

So far, p53 has been characterized in several mammalian species where its biology follows more or less what is known in mice and humans [10]. However, selective pressures associated to certain life styles may modify the properties of p53 and its signaling pathway. For example, the Israeli mole rat (*Spalax*) who lives in hypoxic conditions does not activate p53 in response to hypoxia [11,12]. In addition, ground squirrels have lower levels of p53 in their nucleus during hibernation in comparison with animals during the hot summer season [13]. The discovery of p53 in model organisms such as *Drosophila *and *C. elegans *revealed that p53 evolved in connection with the regulation of apoptotic pathways in response to DNA damage [14,15]. Since these animals do not develop tumors, the p53 tumor suppressor functions probably evolved later in evolution. Unfortunately, little is known about p53 biology in non-mammalian vertebrates, where tumor suppressor functions may play an important role. P53 has been characterized in zebrafish where its biology and tumor suppressor functions seem to be similar to mammals [16]. In contrast, a recent study in rainbow trout cells seems to indicate that p53 is not up-regulated following exposure to chemotherapeutic drugs [17]. Altogether, these studies reveal a surprising adaptive capability of the p53 system to different life styles and suggest that more interesting variations are still to be found in nature.

In the present study, we used the Mexican salamander, axolotl (*Ambystoma mexicanum*), which is a diploid urodele amphibian with: a remarkable longevity (up to 25 years in captivity) [18]; extensive regenerative capacities that have been known for centuries [19-22]; and resistance to tumor induction [23,24]. Urodele amphibians have been used for tissue regeneration and cancer resistance studies for which many authors have established links between the ability to regenerate and the resistance to cancer observed in these animals [23-28]. Since p53 is a tumor suppressor that can influence the rate of aging and modulate regeneration in both mammals and Drosophila imaginal discs [9,29,30], we reasoned that a long living animal such as the axolotl may teach us lessons on how the p53 system is properly modulated to ensure a long life span, tissue regenerative capacity and efficient tumor suppression. Here, we show that p53 signaling is required for limb regeneration, by using a specific pharmacological inhibitor that has been shown to block p53 signaling in both mammals and zebrafish [31,32] and we also present the cloning and initial characterization of p53 in axolotls. We discuss the unique properties of the axolotl p53 sequence compared to both short living (mice, flies) and long living species (moles, humans). By understanding the evolutionary changes that have occurred in genes such as p53, we may be able to understand better why mutations in this gene cause neoplasia [16] and how it can mediate tissue regeneration [33].

## Results

### Requirement of p53 signaling for limb regeneration

**Figure 1:**
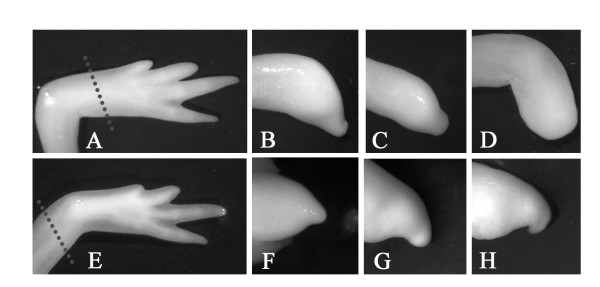
**Effect of pifithrin-α on limb regeneration**. (A & E) Controls treated daily with DMSO. (B-D & F-G) Pifithrin-α treated animals (5 μM pifithrin-α, added freshly diluted everyday). Limbs in panels A-D were amputated distally in the middle of the zeugopod and limbs in panels E-G were amputated proximally through the middle of the stylopod (see dotted lines in panels A & E for amputation levels).

We have used the small molecule inhibitor pifithrin alpha (pifithrin-α) to block the action of p53 during the process of limb regeneration in axolotls [31,32,34-36]. Pifithrin-α was administered at the time of amputation, directly in the water that axolotls are kept in, at a final concentration of 5 μM (a concentration demonstrated to be specific for p53 inhibition [35]). Control animals were administered a volume of DMSO corresponding to the volume for pifithrin. Limb regeneration was monitored at regular intervals until the control treated limbs had completely regenerated (25 days). The pifithrin-α treated animals displayed a significant inhibition in the regenerative process as seen in figure 1. Animals treated with pifithrin-α displayed normal growth compared to control treated size matched animals which indicates that the effects of pifithrin-α were not due to general toxicity (table-1).

**Table 1:** Growth of control and pifithrin-α treated animals during limb regeneration

Animal	Treatment	Lenght of the pre-amputated animals	Lenght of the animals 24 days post-treatment and amputation	Growth Ratio (pre-amp/post-amp)
1	Control DMSO	3.5 cm	4 cm	0.88
2	Control DMSO	3.5 cm	3.9 cm	0.9
3	Control DMSO	3.0 cm	3.5 cm	0.86
4	Pifithrin-α 5 μM	3.0 cm	3.5 cm	0.86
5	Pifithrin-α 5 μM	3.2 cm	3.7 cm	0.86
6	Pifithrin-α 5 μM	3.0 cm	3.4 cm	0.88

no significant difference in growth observed between control and pifithrin treated animals (p > 0.05)

### Axolotl p53 protein, sequence comparison and phylogeny

**Figure 2:**
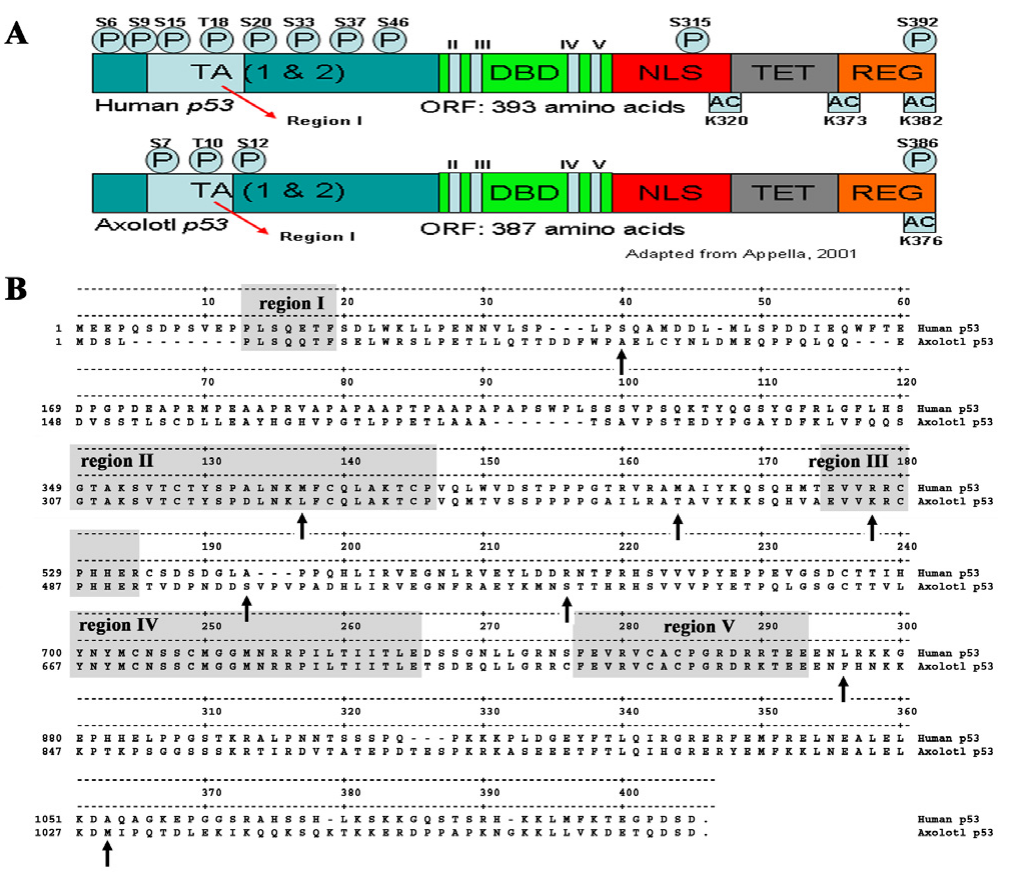
**Comparison between Human and axolotl p53 important domains, regions and residues**. (A) Schematic structure of p53 protein (adapted from Appella, 2001 [71]): TA, Transactivation Domain; DBD, DNA Binding Domain; NLS, Nuclear Localisation Signal; TET, tetramerisation domain; REG, Regulatory domain; Regions I-V, highly conserved regions. Lysine (K), serine (S) and threonine (T) residues implicated in post-translational modifications are indicated. The protein domains depicted in the diagrams are not to scale. (B) Sequences alignment of human and axolotl p53 proteins. The conserved regions I to V are highlighted and many changes between the axolotl and human sequence are identified (arrows). See table-2 for a complete list of changes associated with mutations in the human protein.

We cloned the axolotl full-length p53 cDNA by a combination of PCR and cDNA library screening approaches. The axolotl p53 protein (GENBANK accession number DQ848588) has an open reading frame of 387 amino acids with significant sequence similarity to the human p53 protein (Figure 2A–B). Functional domains of the axolotl p53 protein that correspond to the human protein sequence can be readily identified, namely the N-terminal domain (transactivation domain (residues 1–53 of the human protein), proline rich domain [37] (residues 60–92), the DNA binding domain (residues 80–301), the tetramerization domain (residues 316–347), and the C-terminal regulatory domain (residues 352–388) [38].

The critical residues for protein stability and protein-protein interactions in the N-terminus corresponding to serine 15 and serine 20 are conserved. Since these residues are the target of p53 regulation by phosphorylation in response to DNA damage, they suggest that axolotl p53 is also activated by DNA damage inducing stress. The residues F19, W23 and L26, important for the binding of Mdm2, are also present [39]. Furthermore, regions I of the transactivation domain as well as regions II-V of the DNA binding domain (II-IV-V bind to SV40 T Antigen) can be easily distinguished [40] (Figure 2B). Several residues present in the tetramerization domain of p53 proteins such as: E326, G334, E339, M340, N345, L348, and L350 are conserved as well (Figure 2B) [41]. Finally, it is possible to observe the NLS (Nuclear Localization Signal, residues 296–314) and the NES (Nuclear Export Signal, residues 332–343) (Figure 2B) [42].

We compared the sequence of the axolotl protein with the database of p53 mutants [43]. We found 38 amino acid changes in the axolotl protein that corresponded to mutations present in human cancers or affect protein stability or modify sites of importance for post-translational modifications (table-2 which also contains scorecons [44]). They include the sites frequently mutated in human cancers: T155, V157 and R283. Noticeable the changes T155A and V157L are common to human p53 mutant proteins and axolotl p53. On the other hand, the residue R283 was found substituted for Lysine in axolotl, a change that abolished DNA binding for the human protein [45].

**Table 2:** Analysis of the changes in aa of the axolotl p53 protein compared to human p53

Human codon	# of mutations reported at this site	Axolotl codon	# of mutations identical to the axolotl sequence	temperature sensitive mutations	scorecons	aa changes at this position S/N/X
**S 37 : TCC**	**4**	**A : GCA**	**0**		**0.35**	**+/+/+**
**M 133 : ATG**	**49**	**L : CTC**	**4**		**1**	**+/+/+**
T 150 : ACA	38	P : CCG	1		0.39	-/+/+
T 155 : ACC	128	A : GCA	12	I, N, P	0.02	-/+/+
V 157: GTC	267	L : CTC	10		0	-/-/+
***M 160 : ATG***	***45***	***T : ACT***	***0***	***I, K, R, T***	***0***	-/+/+
I 162 : ATC	59	V : GTG	7		0	-/+/+
**R 174 : AGG**	**78**	**K : AAA**	**12**		**0**	**+/+/+**
S 185 : AGC	23	P : CCC	0		0.06	-/+/+
D 186 : GAT	23	N : AAT	5		0.01	-/+/+
G 187 : GGT	42	D : GAC	5		0.01	-/+/+
L 188 : CTG	14	D : GAT	0		0	-/+/+
***A 189 : GCC***	***41***	***S : TCT***	***1***	***P, S***	***0***	-/+/-
P 191 : CCT	55	A : GCC	0		0	-/+/-
Q 192 : CAG	112	D : GAT	0		0	-/+/+
L 201 : TTG	33	F : TTC	6		0.03	-/+/-
L 206 : TTG	20	K : AAG	0	F	0.01	-/+/+
D 207 : GAT	23	M : ATG	0		0	-/+/+
D 208 : GAC	49	N : AAT	6	A, V	0	-/-/-
**R 209 : AGA**	**90**	**S : TCG**	**1**		**0.01**	**+/+/+**
F 212 : TTT	47	H : CAT	0	V	0	-/+/+
P 222 : CCG	32	T : ACG	1		0.02	-/+/+
E 224 : GAG	54	Q : CAG	0		0	-/+/+
V 225 : GTT	27	L : CTC	1		0.03	-/-/-
D 228 : GAC	51	G : GGA	6		0.01	-/-/+
I 232 : ATC	67	V : GTG	6	F, M	0	-/+/+
H 233 : CAC	38	L : CTT	2		0	-/+/+
D 259 : GAC	90	T : ACT	0		0.01	-/+/+
G 262 : GGT	46	E : GAG	0		0	-/-/-
N 263 : AAT	24	Q : CAG	0		0.02	-/+/+
N 268 : AAC	25	R : CGC	0	I, T	0.01	-/+/+
S 269 : AGC	46	C : TGC	4	G, I, R	0	-/+/+
R 283 : CGC	126	K : AAG	0	P	0	-/+/-
**L 289 : CTC**	**32**	**F : TTT**	**5**	**P**	**1**	**+/+/+**
F 338 : TTC	7	Y : TAT	0	S	0.21	-/+/+
E 343 : GAG	7	K : AAG	0	G	0.01	-/+/+
**A 353 : GCC**	**1**	**M : ATG**	**0**	**P**	**0**	**+/+/+**
S 376 : TCT	2	P : CCT	0	T	0	-/+/+

**Bold**: aa changes present in axolotl, Spalax (S), Newt (N) and Xenopus (X)

***Italic bold***: aa changes causing p53 to become temperature sensitive

We also compared the sequence of axolotl p53 with that of *Spalax*, the newt, *Xenopus *and the human. We found that R174 present in human is substituted by a lysine in *Spalax*, newt, *Xenopus *and axolotl. K174 in *Spalax *is believed to be linked to a reduced ability of p53 to induce apoptosis [11]. In human tumors, R174 has been found mutated into several different amino acids including lysine [46,47]. In vivo analysis, in a yeast model of p53 transactivation performed at 30°C, showed that p53K174 was able to transactivate several p53 promoter-reporters similar to wild type p53 [48].

Given that R174K mutation was not a unique sequence feature of axolotl p53, we conducted a sequence alignment of the p53 from several vertebrates spanning mammals to fish (human, mouse, rat, Spalax, chicken, axolotl, Xenopus, barbel & zebrafish) to identify the changes in table-2 that are unique to the axolotl p53. We found that the changes T155A, D186N, L188D, D207M & D228G represented positions for which a non-conservative change was present only in the axolotl (additional file 1). Notably the changes A189S, D208N & G262E were unique to the axolotl.

**Figure 3:**
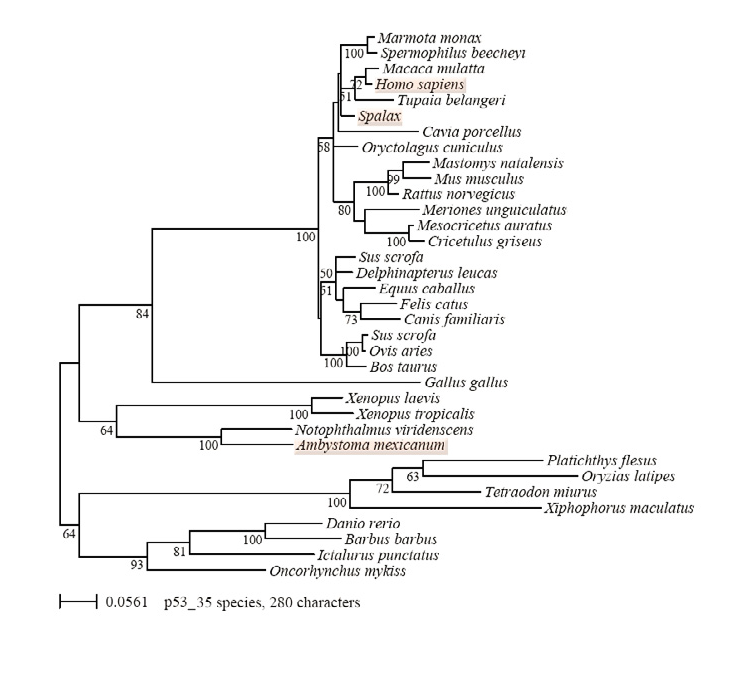
**Phylogenetic tree of p53 protein sequences in vertebrates with bootstrap values**. Maximum likelihood phylogenetic tree based on 35 p53 sequences with 280 amino acid positions inferred by the program Treefinder with a WAG+Γ8 model. Numbers at internal nodes are corresponding to bootstrap support values, obtained in the analysis of 100 replicates using the same program and model of sequence evolution. Due to the dense species sampling within the mammals interesting aspects of mammalian evolution are becoming apparent. There are clear differences in the evolutionary rates among the different groups, indicated by the branch length of the rooted tree, this is especially true for the four Neoteleost fish. There is also an acceleration observed for the mouse-like rodents, with a striking exception represented by the sequence of the mole rat *Spalax*, which is despite the fact of being a rather small rodent even more slowly evolving than the related rabbit (*Oryctolagus*, lagomorph). In fact the only sequences among the tetrapods (amphibians, reptilian and mammalian) that are more slowly evolving than the one from *Spalax *are from the urodeles (axolotl and newts). The primary sequence of salamander p53 is more closely related to the ancestral protein of tetrapod vertebrates than the p53 proteins of any other of the studied groups.

We used our new axolotl sequence and many already published p53 protein sequences from vertebrates to construct a maximum likelihood based phylogenetic tree (35 sequences and 280 unambiguously aligned amino acid positions). The phylogenetic tree of p53 is in general agreement with the known vertebrate phylogeny (Figure 3). Intriguingly both rodents and fishes were clearly divided into two groups in the p53 phylogeny.

The taxonomic distribution is strongly biased in favor of mammalian (22 sequences) and ray-finned fish (eight sequences). The four Neoteleost fish are evolving very rapidly resulting in artefactual basal position. This long branch attraction artefact is induced by the distantly related and rather fast evolving tetrapods. But also in other parts of the tree there are clear differences in the evolutionary rates (although less striking) among the different groups and also between species of the same group, indicated by the branch length of the rooted tree. There is a pronounced acceleration observed for the mice-like rodents (Muroidea), with a clear exception represented by the sequence of the already mentioned mole rat *Spalax*. There also seems to be a rough inverse correlation between the evolutionary rates inferred for the p53 sequences and the longevity of the species i.e. the faster the rate the shorter the life span. This is evident for all mice-like rodents and the guinea pig except *Spalax*. This is further supported by the observation that the rather big and long-living organisms, human and Beluga whale, have some of the shortest branches within the mammals (Figure 3). We decided to test the significance of the differences in the evolutionary rates especially with respect to the model organisms, i.e. human, mice, *Spalax *and axolotl by applying a Relative Rate Test (RRT) implemented in the program RRTree [49]. It is well known that the significance level is strongly influenced by the distance of the outgroup, i.e. the closer the outgroup sequence the better is the sensitivity of the RRT. We decided to compare within the mammals the Euarchontoglires (here Primates, Rodents and Lagomorphs) *Spalax *and Human against the mouse-related group of rodents (six sequences) with the chicken as a distant outgroup and the Beluga whale (Laurasiatheria) as a close outgroup. The RRT were giving the following probabilities (p < 0.05 is significant) for *Spalax *versus mouse-related rodents, Beluga whale p = 0.000 and Chicken p = 0.042 and for human versus mouse-related rodents, Beluga whale p = 0.002 and Chicken p = 0.135. Therefore, the observation that *Spalax *is more slowly evolving is significantly supported by the RRT for both comparisons. However, note the strong dependence of the p-value on the chosen outgroup. For human, only the test with the close outgroup is significantly supported. If we applied the same test to compare the evolutionary rate of axolotl with the one of Xenopus with the fish and amniotes as outgroup, the outcome was not significant (p = 0.310). This is likely due to the fact that the sensitivity of the test is highly reduced in absence of a close outgroup. The phylogenetic analysis shows that the urodele p53 sequences are among the slowest evolving ones and are therefore closer to the ancestral p53 protein of tetrapods. Bayesian inference also yielded similar results (data not shown) which further substantiate the results of the phylogenetic analysis presented in figure 3. Unfortunately, because of the lack of p53 sequences in non-mammalian vertebrates, there were only good conditions for the relative rate within mammals and could therefore not provide reliable information for the evolutionary rate of the axolotl sequence in comparison to other amphibians. Thus, we have to exclude any potential correlation with the longevity of the species until more sequences become available.

#### Axolotl p53 can activate the transcription of human p53 target promoters in human cells

The amino acid substitutions found in the axolotl p53 protein sequence raises questions about its ability to recognize and regulate human p53 target genes. To investigate this issue, we used a Dual-Luciferase Reporter assay in the human p53 deficient cell line H1299. We co-transfected either human or axolotl p53 expressing plasmids with luciferase reporter constructs fused to the human Hdm2 and p21 promoters. We performed these assays at three different temperatures (37°C, 30°C and 25°C) for two main reasons. First, two changes present in the axolotl protein have been reported to cause the human protein to become temperature sensitive (M160T & A189S, Figure 2B and table-2) [50]. Second, the p53 protein from *Xenopus laevis *was shown to be temperature sensitive, and also contains substitutions at M160, L289 and A353 (table-2) which rendered the human protein ts [51].

**Figure 4:**
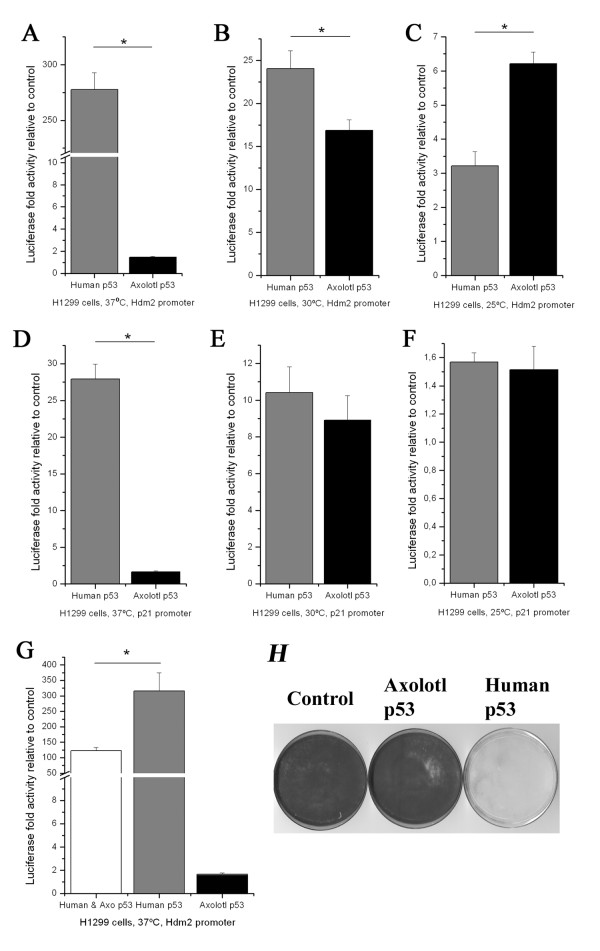
**Activation of Hdm2 and p21 promoters by human and axolotl p53 in H1299 cells**. (A-C) Dual-luciferase assays in H1299 cells with Hdm2 promoter at 37°C, 30°C and 25°C. (D-F) Dual-luciferase assays in H1299 cells with the human p21 promoter at 37°C, 30°C and 25°C. Luciferase activities stimulated by human or axolotl p53 was significantly different than non-p53 controls (at least p < 0.05, data not shown). Error bars are ± s.e.m. human and axolotl p53 luciferase transactivation were significantly different during the same assay using the Hdm2 promoter (A-C) or the human p21 promoter at 37°C (D) (at least p < 0.01). Each assay was performed in triplicate at least 3 separate times. (G) Inhibition of the activation of Hdm2 promoter by the combined expression of human and axolotl p53 in H1299 cells. All luciferase activities were significantly different than non-p53 controls (at least p < 0.05, data not shown). Error bars are ± s.e.m. human + axolotl and human p53 luciferase transactivation were significantly different during the same assay using the Hdm2 promoter at 37°C (p = 0.006). Each assay was performed in triplicate. (H) Growth assays at 37°C on H1299 cells transfected with human or axolotl p53 protein.

Human p53, as expected, stimulated the Hdm2 promoter, at 37°C. However, the axolotl protein was relatively inactive at this temperature (Figure 4A). In contrast, at 30°C, we observed that the human p53 stimulated the Hdm2 promoter by a factor of 24 fold and that the axolotl p53 stimulated a similar increase of 17 fold (Figure 4B). Further reduction of the temperature to 25°C shifted the activity pattern on the Hdm2 promoter. At this temperature the axolotl protein was twice as active as the human p53 protein (Figure 4C). Thus, for the Hdm2 reporter, the human p53 is more active at 37°C and less active at 25°C while the axolotl p53 activity is less active at 37°C and more active at 25°C.

On the p21 promoter luciferase reporter, human p53 stimulated 28 folds the activity at 37°C, 10.5 folds at 30°C and 1.6 folds at 25°C (Figure 4D–F). When the axolotl p53 was assayed, we observed an increase of 1.7 fold at 37°C, an 8.9 folds increase at 30°C and 1.5 fold increase at 25°C. The peak activity of the axolotl p53 on this promoter seems to be near 30°C as opposed to 37°C for the human p53 protein. However, at any given temperature the ability of axolotl p53 to activate the p21 promoter was compromised compared to the Hdm2 promoter. Among the amino acid substitutions of the axolotl protein that may explain this reduced activity for the p21 promoter we noticed the change of Ser 37 to Ala (Table-2). Substitution of Ser 37 for Pro has been detected in a T lymphoblastoid cell line that is drug resistant. This mutant has a reduced activity on both the p21 and the p53 promoter [52].

Next we assayed the human and the axolotl p53 together to activate the Hdm2 promoter luciferase reporter at 37°C. We noted a decrease in the expression of luciferase when the axolotl protein was transfected together with the human protein compared with the human p53 alone (Figure 4G). Hence, the axolotl p53 seems to have a dominant negative effect on the activity of the human p53 on the Hdm2 promoter at 37°C, suggesting that they can interact together.

To have an idea of the functionality of axolotl p53 on a biological assay, we transfected H1299 cells with constructs driving the expression of human p53, axolotl p53 or an empty vector. As expected from the luciferase assays, axolotl p53 did not reduce colony formation of H1299 cells, while human p53 had a dramatic inhibitory effect in this assay (Figure 4H). Contrary to the promoter-reporter assays which were performed 24 h post-transfection, we could not assay p53 functions for colony formation at 30°C and 25°C in these cells because they did not efficiently attach to the plate at those temperatures for the required amount of time to select with puromycin (7 days). Together, these results are consistent with the idea that axolotl p53 is a temperature sensitive protein.

**Figure 5:**
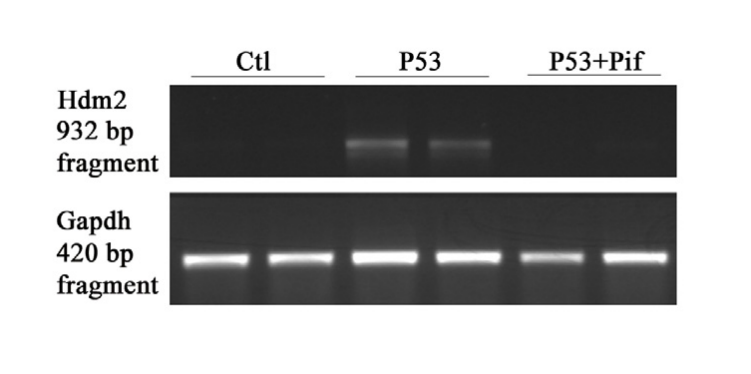
**Activation of endogenous Hdm2 in H1299 cells by the axolotl p53 protein**. Expression of the human p53 target gene (Hdm2) 24 h post transfection in H1299 cells mock transfected and transfected with the axolotl p53 without and with pifithrin-α grown at 30°C. RT-PCRs were also performed with the housekeeping gene glyceraldehyde phosphate dehydrogenase (Gapdh) to control for the amount of total RNA.

The capacity of the axolotl p53 protein to induce endogenous p53 target gene was also tested in the p53 deficient human H1299 cells. The H1299 cells grown at 30° transfected with the axolotl protein had elevated Hdm2 expression (Figure 5). We also took advantage of this assay to confirm the specificity of the pifithrin-α to block the activity of the axolotl p53 protein (Figure 5).

### Axolotl p53 activation in axolotl cells

The axolotl AL1 cell line was used for the subsequent dual-luciferase reporter assay to measure the activity of human p53 and axolotl p53. The assays were done at 25°C since axolotl cells are unable to grow at 37°C or 30°C. The Hdm2 and p21 reporter genes were used for the assays.

**Figure 6:**
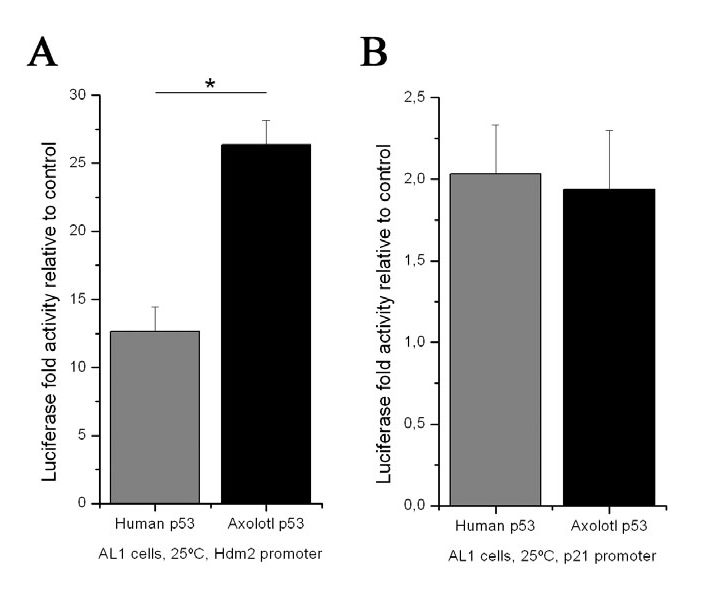
**Activation of Hdm2 and p21 promoters by human and axolotl p53 in AL1 cells**. (A-B) Dual-luciferase assays in AL1 cells with Hdm2 and p21 promoters at 25°C. All Luciferase activities were significantly different from non-p53 controls (at least p < 0.05, data not shown). Error bar ± s.e.m. human and axolotl p53 induced luciferase expression were significantly different with the Hdm2 promoter (p < 0.01). Each assay was performed in triplicate at least 3 separate times.

With the Hdm2 promoter, there was a 12.6 fold increase with human p53 protein and a 26.4 fold increase with the axolotl p53 protein (Figure 6A). Therefore, the axolotl p53 protein activity was significantly more efficient than the human protein at 25°C (p < 0.001) on the Hdm2 promoter in axolotl cells. In contrast, with the p21 promoter, there was a two-fold increase with the human p53 and 1.9 fold increase with axolotl p53 protein (Figure 6B). Again, it seems that the DNA binding specificity of the axolotl protein is different than the human counterpart.

### DNA damage and activation of axolotl p53

**Figure 7:**
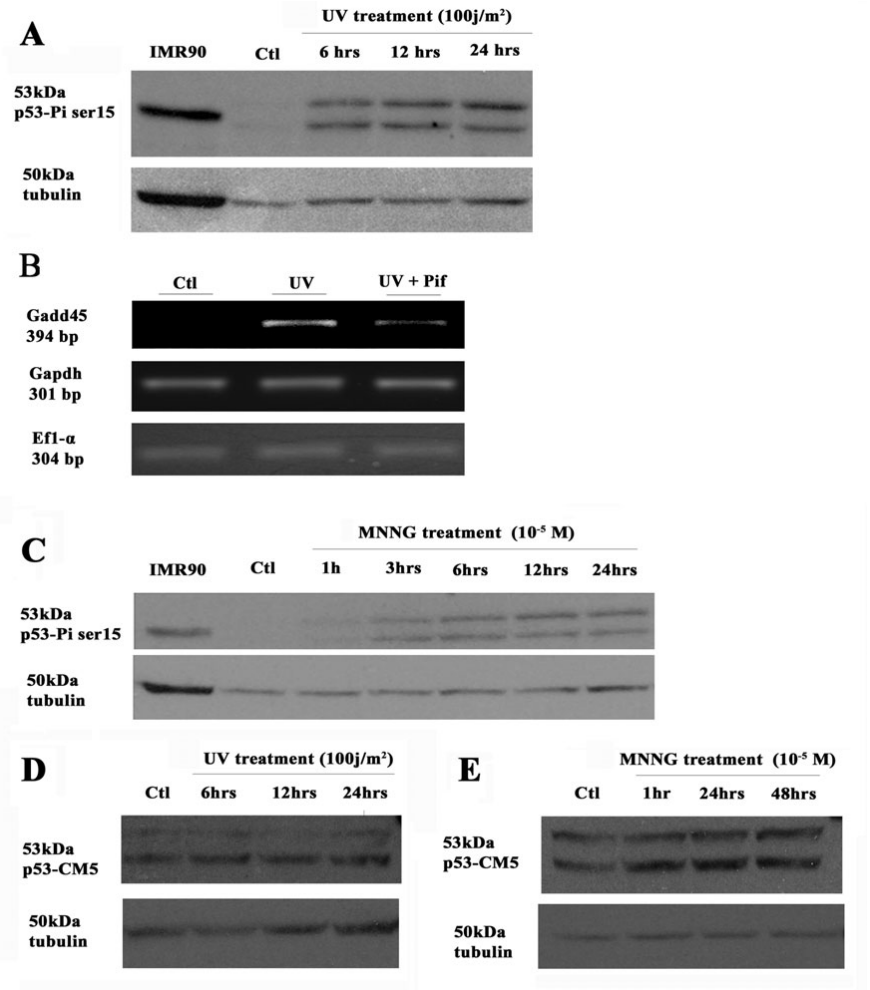
**Detection of p53 protein in AL1 cells**. (A & C) Western blot analysis of phospho-ser15 p53 in AL1 cells exposed to UV or treated with MNNG. (B) RT-PCR analysis of p53 target gene, Gadd45 (a p53 target gene cloned in axolotl [57, 58]), in control treated axolotl AL1 cells, cells exposed to UV (6h post-irradiation) and cells exposed to UV & treated with pifithrin-α. Both Gapdh and Ef1α were used as controls to demonstrate that the effects of UV and UV plus pifithrin-α were specific for Gadd45. (D-E) Western blot analysis of total p53 protein (CM5 antibody) on AL1 cells exposed to UV or treated with MNNG.

Since p53 activity has been shown to be modulated by close to 50 post-translational modifications on the protein itself, we decided to look at a specific phospho-serine residue following different treatments. We used the axolotl cell line AL1 [53] to assess the effect of ultraviolet irradiation on axolotl p53 levels and phosphorylation. Western blots were performed against phospho p53 ser-15 on axolotl AL1 cells exposed to UV radiation or the alkylating agent (N-methyl-n-nitro-n-nitrosoguanidine; MNNG) which causes DNA breaks [54,55]. The cells exposed to UV showed an increased phosphorylation of p53 at serine 15 in comparison to the non-exposed control cells (Figure 7A). We noticed that anti-p53 antibodies recognized two bands that followed the same induction pattern in response to DNA damage. These bands may represent alternatively spliced p53 isoforms as described in mammals [56]. The availability of the axolotl ortholog for Gadd45 allowed us to look at the induction of an endogenous p53 target gene following UV irradiation of axolotl cells [57,58]. The capacity of pifithrin-α to block p53 signaling was also tested. Axolotl cells exposed to UV irradiation showed an up-regulation of Gadd45 expression, which was significantly inhibited in the presence of pifithrin-α (Figure 7B). Treatment with MNNG (10^-5^M) also increased p53 phosphorylation at serine 15. The signal increase for phospho p53 ser-15 was detectable one hour after drug application and up to 24 hours later (Figure 7C). On the other hand, an antibody against "total" p53 (CM5) was used on Western blot analysis to determine total protein levels in AL1 cells exposed to UV and to MNNG and showed no increase in expression (Figure 7D–E). These results suggest that the axolotl p53 activity is regulated by post-translational modifications in cells exposed to UV and MNNG.

## Discussion

The p53 sequences of lower vertebrates (amphibians and fishes) that have been identified to date are quite divergent from their mammalian counterparts. However, they all conserve the basic domain organization described for human p53. We investigated the axolotl p53 protein for various reasons: First, this is a well characterized lower vertebrate that has been used as a model organism for over 100 years; second, axolotls display a remarkable resistance to cancer [23,26,27,59,60]; and third axolotl is long living, up to 25 years in captivity [18]. We are interested to determine to which extent p53 biology in axolotls is linked to their remarkable ability to regenerate lost tissues and their cancer resistance.

To address these questions, we tested whether p53 signaling was required for limb regeneration. The administration of pifithrin-α blocked the process of limb regeneration therefore providing evidence that p53 signaling is required for regeneration in axolotls. These results are consistent with the newly reported role of p53 requirement for regeneration of both axons in mice and imaginal discs in drosophila [29,30]. Then, we cloned the axolotl full-length p53 sequence and compared it to that of other species, in particular human, and the naked mole rat *Spalax*. We found an overall conservation of the p53 functional domains as described for mammals. However, we also found interesting amino acid changes, many of which are unique to the axolotl (see Additional file 1).

First, the axolotl p53 was largely inactive at 37°C, which can be explained by the presence of two changes in amino acids (M160T & A189S) that have been shown to cause the human protein to become temperature sensitive [50]. Also, the residue at position R174 in the human sequence is changed to lysine in the axolotl. We noticed a similar substitution in *Spalax*, Xenopus and the newt. In the model of human tetrameric p53 bound to the DNA, R174 does not contact the DNA and it was proposed that it interacts with R181 in an adjacent monomer [11]. The neighboring R175 mediates interactions between the loops that contact the DNA and may stabilize the core structure of the DNA binding domain [61]. Mutation in R175 to histidine makes DNA binding by p53 temperature sensitive [62]. Mice expressing R175H-p53 revealed not only a dysfunctional protein but also gain of function activities such as the ability to have a dominant negative effect on wild type p53 and other p53 family members such as p73 [63,64].

Another interesting change is the T155A which we found is unique to the axolotl (Additional file 1). This position is one of the most frequently mutated in human cancers. However, the transactivation ability of the T155A protein on several promoters is not significantly disabled by this mutation and it is doubled on the Noxa promoter [48]. In addition, phosphorylation of T155 by the COP9 signalosome targets p53 for degradation by the proteasome [65] and disruption of the COP9 signalosome Csn2 subunit results in a hyperactivation of p53 [66]. These results suggest that the axolotl protein may be resistant to degradation allowing the protein to accumulate and therefore could confer cancer resistance. In agreement with this hypothesis, mice expressing extra-copies of p53 are also cancer resistant [67].

We also studied the response of axolotl p53 to DNA damaging agents such as UV light and an alkylating agent. These results clearly indicate that p53 is activated following UV irradiation in axolotl cells as reported in human cells [68]. Also, our time course experiments showed an increase in phospho-ser15 p53 in axolotl cells treated with 10 μM of MNNG. Our data clearly confirms that p53 is activated in salamanders through similar mechanisms as described for mammalian cells under similar conditions.

Multiple p53 codons are found mutated in human cancer and many of them have been tested for transactivation, induction of growth arrest or apoptosis. However, combinations between different individual mutations have not been systematically studied, mainly because their number is obviously too large. The discovery of a natural p53 variant containing a combination of mutations found in human cancers is a rich source of structural data for fundamental studies about p53 and represents a window to understand how certain selective pressures modify the structure and the function of p53. For example, the p53 protein from *Spalax *was unable to regulate several promoters of pro-apoptotic p53 targets and it was suggested that this trait is an adaptation to hypoxic life [11]. It is interesting to speculate that changes we have found in axolotl are the result of selective pressures linked to the life style of this animal, including its ability to regenerate lost tissues.

In conclusion, our results present for the first time the p53 system in axolotls and open the way to study whether this interesting protein is linked to the remarkable traits that have evolved in these amphibians. Further characterization of p53 in these animals may shed light into the evolutionary adaptations of the p53 tumor suppressor pathways to different physiological conditions and the potential relationships with the aging/cancer process.

## Methods

### Animal procedures

Axolotls (*Ambystoma mexicanum) *were purchased from the Axolotl colony (Ambystoma Genetic Stock Center, Lexington KY). Larvae were maintained at 20–22°C in 40% Holtfreter' solution. Animals (2.5–4 cm) were anaesthetised in 0.1% MS222 solution for amputations through both the upper (proximal) and lower (distal) part of the forelimbs to induce regeneration. Pifithrin-α (Sigma-Aldrich) was dissolved in 100% DMSO at a concentration of 5 mM. Pifithrin-α was administered by adding directly to the Holtfreter's solution to the desired concentration (at least 9 animals were treated with 5 μM); treatment was started at the time of amputation. The control animals were treated with the same 100% DMSO that was used to dissolve the pifithrin-α. All solutions were changed daily until the controls had completely finished regenerating their limbs (25 days post-amputation). Animals were euthanized at the end of the experiment.

### Cell lines

H1299 (*p53*-null lung cancer cell line) and IMR90 (human Caucasian fetal lung fibroblast) cell lines grown in culture were maintained at a constant temperature of 37°C with 5% CO_2 _in Dulbecco's Modified Eagle Medium (DMEM with 1× L-glutamine, 1× penicillin-streptomycin and 10% Fetal Bovine Serum) (Gibco, Invitrogen, Carlsbad, CA). Axolotl cells (AL1) were obtained from Dr. S.V. Bryant and Dr. D.M. Gardiner at the University of California Irvine. AL1 cells grown in culture were maintained at a constant temperature of 25°C without CO_2 _in Leibovitz's L-15 medium (L-15 60.0% with 1× L-glutamine, 1× antibiotic-antimycotic, 1× insulin-transferrin-selenium and 5% Fetal Bovine Serum) (Gibco, Invitrogen, Carlsbad, CA).

### Cloning and sequence analysis of axolotl p53 cDNA

Partial axolotl p53 cDNA sequences were cloned by RT-PCR using degenerate primers [partial axolotl p53 forward (DFPROP53)] 5'-GG(A/C/G/T)(C/T)T(A/C/G/T)GC(A/C/G/T)AA(A/G)ACITG(C/T)CC-3' and [partial axolotl p53 reverse (DRPROP53)] 5'-G(G/T)(A/G)TTCAT(G/T)CC(G/T)CCCAT(A/G)CA-3'. To obtain the full length *p53 *gene, we performed a screening of Salamander Larvae Lambda cDNA Library (Stratagene, La Jolla, CA), with the partial p53 cDNA sequence obtained. The open reading frame of the p53 protein was identified after sequencing several cDNA isolates from the library (4 different isolates). Two primers with a restriction site on each side (5'Xho1 and 3'BstX1) were made to facilitate the cloning of full length axolotl p53 in the FG12/CMV vector (M. Soengas, Ann Arbor). Full length p53 cDNA was subsequently amplified by PCR using [axolotl p53 forward XHO (APFXHO)] 5'-GAGCCTCGAGGAATCGGAAACCACCATGGA-3' and [axolotl p53 reverse BSTX (APRBSTX)] 5'-GCGCCCATCTTTATGGGAATACAGGCACCATTGCAG-3' primers. PCR reactions were performed using Taq DNA polymerase (Invitrogen, USA). All PCR products were purified by Rapid gel extraction (Gibco), cloned into the pCR 4-TOPO vector (Invitrogen, USA) and sequenced by the McGill Genome Sequencing Centre with M13R and M13F primers. Resulting sequences were assembled using SeqManII (DNASTAR Inc., USA). All sequences were analysed on the NCBI BLAST database (axolotl p53 GENBANK sequence accession number: DQ848588).

### Transfection of human cells and dual luciferase reporter assay

H1299 cells were transfected at a confluence of 90% in 6 wells plates, with 2.5 μg of the firefly luciferase reporter plasmid (p21 waf-1-luc or Hdm2 in pGL3 vector; Promega), 0.5 μg of a plasmid expressing either human p53 (pLPChp53 containing the CMV promoter), axolotl p53 (FG12/CMV-Ap53) or FG12/CMV as control. A plasmid expressing *Renilla *luciferase under the β-globin promoter was co-transfected (0.5 μg) to normalize the results of the assays. Transfection was done with lipofectamine 2000 (Invitrogen, U.S.A.) according to the manufacturer's instructions. H1299 cells were incubated at different temperatures (37°C, 30°C or 25°C) following transfection. 24 hours after transfection H1299 cells were rinsed with Hank's buffer and harvested with trypsin (Invitrogen, CA). They were resuspended in 200 μl of fresh passive lysis buffer (Promega), vortexed, frozen in liquid nitrogen and stored at -80°C. Luciferase assay was performed following the standard protocol for the Dual-Luciferase^® ^Reporter Assays System (Promega, Madison, WI). The assays were read on a Fusion α-FP luminometer (Packard Instrument Company, Inc., Meriden, CT). For the experiments combining the human and axolotl p53 we used 0.5 μg of plasmids expressing human and axolotl p53 (or FG12/CMV for control), 2.5 μg of the firefly luciferase reporter plasmid (Hdm2 in pGL3 vector) and 0.5 μg of plasmid expressing *Renilla *luciferase under the β-globin promoter.

### Electroporation of axolotl cells and dual luciferase reporter assay

An electroporation protocol was developed to transfect the AL1 cells for the Dual Luciferase^® ^Reporter Assay. Cells were co-transfected with 5 μg of the firefly luciferase reporter plasmid (p21 waf-1-luc or Hdm2 in pGL3 vector; Promega) and with 1 μg of a plasmid expressing either human p53 (pLPChp53), axolotl p53 (FG12/CMV-Ap53) or empty FG12/CMV as control. The same plasmid expressing *Renilla *luciferase under the β-globin promoter (1 μg) was used to normalize the results of the assays. The transfection was performed using a Gene-Pulser Xcell Eukaryotic System (Bio-Rad) generating a single electrosquare pulse of 250 volts during 35 milliseconds, in a 4 mm cuvette (VWR) containing approximately 60 000 AL1 cells resuspended in 100 μl of ice cold 0.7× PBS. 48 hours after electroporation, axolotl cells were rinsed twice with 0.7× PBS, lysed with 250 μl of fresh 1× passive lysis buffer and placed on a shaker with gentle agitation for 20 minutes. The cells were collected, frozen in liquid nitrogen and stored at -80°C. Luciferase assays were performed as described above.

### RT-PCR

Total RNA was extracted from either H1299 cells of axolotl AL1 cells using Trizol reagent (Invitrogen, Carlsbad, CA). Reverse transcription reactions were done at 50°C using Superscript II reverse transcriptase (Invitrogen). The cDNA fragments encoding for the human Hdm2 (Genbank # CO783557) and the axolotl Gadd45 (Genbank # NM_002392) were amplified by RT-PCR from total RNA with the following primers: Hdm2 forward CAGCTTCGGAACAAGAGACC, reverse GAAGCCAATTCTCACGAAGG; Gadd45 forward CGTGCACCTTACTTGGGACT, reverse ATGTCATTGTCGCAGCAAAA. Gapdh and EF1-α were used as control PCR using the following primers: human Gapdh forward ACCACAGTCCATGCCATCAC, reverse TCCACCACCCTGTTGCTGTA and for the axolotl EF1-α forward AACATCGTGGTCATCGGCCAT, reverse GGAGGTGCCAGTGATCATGTT, and Gapdh forward GACAAGGCATCTGCTCACCT and reverse ATGTTCTGGTTGGCACCTCT. RT-PCR reactions were performed on at least 3 separate RNA preparations.

### Colony assays

The H1299 cells used for the colony assays were transfected with lipofectamine 2000 according to the manufacturer's instructions. The pLPChp53, the FG12/CMV-Ap53 or the FG12/CMV empty vectors (12 μg) were co-transfected with a puromycin resistant vector (12 μg, pBabe-lacZ) for colony selection. The colony assay was performed 1 week post-transfection at 37, 30 and 25°C in DMEM. The selection was made with 1 μg/ml of puromycin (Sigma-Aldrich, St-Louis, MO). Colonies were colored with crystal violet (Sigma-Aldrich, St-Louis, MO).

### Immunoblots

AL1 cells used in western blotting were exposed to UV (100 j/m^2^) using a XL-1000 UV Crosslinker (Spectronics Corporation, NY, USA). AL1 cells were harvested 6, 12 and 24 hours post-irradiation. Total proteins of axolotl cells were extracted by sonication in sodium dodecyl sulphate (SDS) sample buffer. AL1 cells used in western blotting were also treated with MNNG (1-methyl-3-nitro-1-nitrosoguanidine, Sigma-Aldrich, St-Louis, MO), during 1, 3, 6, 12 and 24 hours, then harvested post-treatment. Total proteins where prepared by sonication as mentioned above. The protein quantification was done using the Bradford technique [69]. 40 μg of cell proteins were denatured by boiling and loaded on 10% polyacrylamide-SDS gels following the Laemmli method [70]. Proteins were then transferred onto polyvinylidene difluoride membranes (Immobilon-P, Millipore, Bedford, MA). Anti-Pp53 ser15 (#9284, Cell Signaling technology, MA) and Anti-p53 CM5 (NCL-p53-CM5p, Novo Castra laboratory, UK) rabbit affinity isolated antibodies were used to detect axolotl p53. Immunodetection of primary antibodies was visualized using the ECL Western blotting kit (Amersham Pharmacia, Amersham, United Kingdom) or Lumi-light Western Blotting Substrate (Roche) following manufacturer's guidelines. Anti-α-tubulin (Tubulin-α Ab-2 clone DM1A, Neo Marker distributed by Medicorp, Qc) was used as loading control. IMR90 cells were used as a positive control for normal expression of p53 protein.

### Sequence handling and phylogenetic analyses

All protein sequences were retrieved from NCBI GenBank after identification by a BLASTP search, with the salamander p53 protein sequence as query (except for the newt (*Notophthalmus viridescens) *p53 sequence which was the generous gift from Dr J. Brockes University College London). Clustal V was used to create the original alignment that was manually further optimised and redundant sequences were discarded using the edit option of the MUST package. G-blocks was used to eliminate variable regions of the alignment. The maximum likelihood phylogenetic tree was inferred by the program Treefinder using the WAG matrix of amino acid replacement under the assumption of four discrete gamma-distributed rates. The statistical robustness of internal nodes was estimated by the bootstrap method, based on 100 replicates using the same program and model as before. The consensus tree of these 100 bootstrap replicates was generated with the consense option of the PHYLIP package. The bootstrap support values given in percent are indicated on the corresponding nodes.

### Statistics

Statistics for the Dual Luciferase Reporter Assay System were performed with SysStat^® ^(Systat Software Inc, USA). One-sample t-test was used to calculate the p-value between control and experimental assays. A two-sample t-test was used to calculate the p-value between experimental assays in same condition. Graphics were made with Origin^® ^(OriginLab Corporation, USA).

## Competing interests

The author(s) declares that there are no competing interests.

## Authors' contributions

The manuscript was written jointly by EV, GF, and SR. EV cloned the axolotl p53 cDNA and did the experiments helped by OM for the western blots of cells exposed to UV and FAM for the colony survival assays; HB performed the phylogenetic tree and related analyses. All authors read and approved the final manuscript.

## Supplementary Material

Additional file 1 Analysis of the changes in aa of the axolotl p53 protein compared to the p53 proteins of multiple vertebrates. This is a supplement to table-2 where we show that 38 aa positions known to be mutated in p53 from human cancers are changed in the axolotl as well. Most of these positions are also changed in the p53 from other vertebrates, however we highlighted in yellow positions that are only changed in the axolotl at aa A189S, D208N & G262E. We also highlighted in blue the positions where only the axolotl showed a non-conservative aa change (T155A, D186N, L188D, D207M & D228G). The data presented in this figure was generated by the pair-wise alignment of the human p53 protein sequence with the protein sequence of each organism. The position number refers to the aa position in the human p53 protein. Gaps in aa sequence alignment are represented by a star (*). Click here for file
