# Supplementary figures and images for: Urodele p53 tolerates amino acid changes found in p53 variants linked to human cancer
Source: BMC Evol Biol. 2007 Sep 28;7:180. doi: 10.1186/1471-2148-7-180 (PMC2072957; doi:10.1186/1471-2148-7-180)

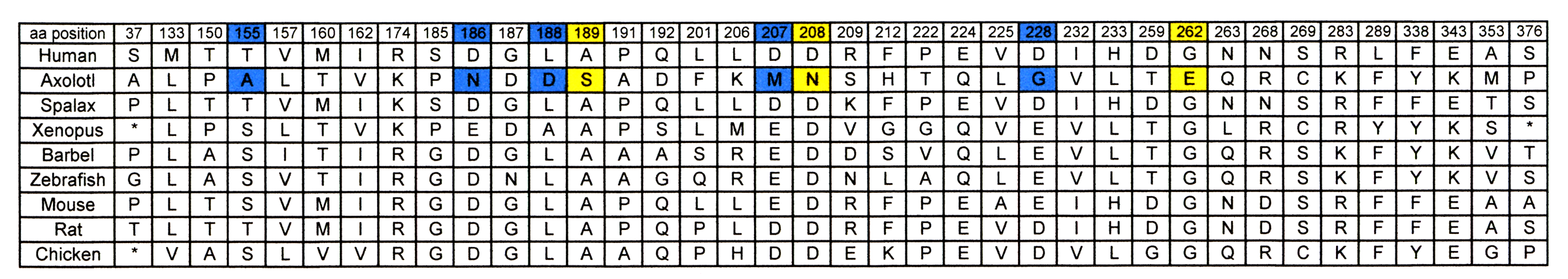

Supplement: Additional file 1 — Analysis of the changes in aa of the axolotl p53 protein compared to the p53 proteins of multiple vertebrates. This is a supplement to table-2 where we show that 38 aa positions known to be mutated in p53 from human cancers are changed in the axolotl as well. Most of these positions are also changed in the p53 from other vertebrates, however we highlighted in yellow positions that are only changed in the axolotl at aa A189S, D208N & G262E. We also highlighted in blue the positions where only the axolotl showed a non-conservative aa change (T155A, D186N, L188D, D207M & D228G). The data presented in this figure was generated by the pair-wise alignment of the human p53 protein sequence with the protein sequence of each organism. The position number refers to the aa position in the human p53 protein. Gaps in aa sequence alignment are represented by a star (*). [file 1471-2148-7-180-S1.tiff]
